# Effects and adaptation of high-altitude hypoxia on lipid metabolism: mechanisms and health implications

**DOI:** 10.3389/fphys.2026.1821632

**Published:** 2026-05-01

**Authors:** Yu Fan, WeiWei Guo, LiXin Yang, HaiQi Xu

**Affiliations:** 1 Clinical Medical College, Qinghai University, Xining, China; 2 Department of Endocrinology, Qinghai Provincial People’s Hospital, Xining, China

**Keywords:** blood lipids, high-altitude hypoxia, lipid metabolism, metabolic adaptation, metabolic syndrome

## Abstract

High-altitude environments impose substantial metabolic constraints on human physiology, with oxygen limitation driving profound alterations in lipid regulation. This narrative review synthesizes current evidence on the context-dependent effects of high-altitude hypoxia (HAH) on systemic lipid metabolism across high-altitude native populations and lowlanders exposed to altitude, with particular attention to Tibetan and Andean settings. Acute hypoxic exposure is characterized by rapid lipid mobilization, enhanced glycolytic flux, suppressed lipoprotein lipase activity, and dynamic fluctuations in circulating lipids. In contrast, acclimatization and long-term adaptation are associated with a shift toward more oxygen-efficient substrate utilization, often accompanied by reduced fatty-acid oxidation, improved insulin sensitivity, and population-specific changes in lipid profiles. However, in non-acclimatized or metabolically vulnerable individuals, sustained HAH exposure may also promote dyslipidemia, inflammation, and adverse cardiometabolic effects. By integrating evidence on HIF-mediated metabolic reprogramming, neuroendocrine regulation, genetic adaptation, and the microbiota–bile acid axis, this review highlights the heterogeneity of lipid responses to hypoxia and discusses their implications for cardiometabolic risk, preventive medicine, and future altitude-related metabolic research.

## 1 Introduction

High-altitude environments (altitude ≥ 2,500 m) are characterized by hypoxia, low temperatures, and intense ultraviolet radiation, each of which can exert profound influences on multisystem metabolism (Pooja et al., 2021). Beyond the well-recognized structural and functional remodeling of the respiratory and hematologic systems, hypoxia-driven regulation of energy metabolism—particularly lipid metabolism—has become a major research focus. Following ascent to high altitude, body weight commonly decreases, and the magnitude of weight loss tends to increase with higher altitude and longer exposure duration. Proposed mechanisms include an elevation in basal metabolic rate and increased activity-related energy expenditure under high-altitude hypoxia (HAH), accompanied by reduced appetite and lower energy intake. Activation of hypoxia-inducible factor-1α (HIF-1α) can upregulate appetite-suppressing hormones such as leptin, thereby inhibiting food intake (Pramsohler et al., 2022). In parallel, acute hypoxia enhances sympathetic nervous system activity, promoting lipid mobilization and lipolysis to increase energy availability (Pérez-Regalado et al., 2024). Accordingly, during the early phase of high-altitude exposure, the organism tends to rapidly mobilize adipose stores to compensate for an energy deficit.

Consistent with these systemic observations, cellular hypoxic responses initiate metabolic reprogramming through hypoxia-inducible factor (HIF) signaling, characterized by upregulated glycolytic capacity and suppression of mitochondrial oxidative metabolism to reduce oxygen consumption (Murray et al., 2018). This adaptive shift can improve ATP yield per unit oxygen consumed; however, because glycogen and glucose reserves are limited, the oxygen-sparing advantage of a predominantly glycolytic strategy is difficult to sustain over time. Therefore, as exposure prolongs, fatty-acid oxidation remains indispensable for sustained energy supply. Notably, fatty-acid oxidation requires more oxygen than glucose oxidation for ATP synthesis; thus, a preferential reliance on glucose can be interpreted as an oxygen-conserving fuel strategy under hypoxic constraint (Ge et al., 2012). Overall, high-altitude hypoxia drives a redistribution of energy substrate utilization, with increased glucose oxidation and a relative suppression of fatty-acid oxidation to accommodate reduced oxygen availability (Pramsohler et al., 2022).

In addition, hypoxia may modulate host lipid metabolism via the gut microbiota. Acute high-altitude exposure can disrupt bile acid metabolic homeostasis (Bai et al., 2025), whereas prolonged hypoxia may increase the proportion of anaerobic bacteria and enhance the production of short-chain fatty acids (SCFAs), which may contribute to maintaining energy metabolic homeostasis and facilitating adaptation. In a study of hypoxia-tolerant high-altitude rodents, hypoxia markedly altered gut microbial composition and the bile acid pool, thereby influencing host lipid metabolism through the farnesoid X receptor (FXR) pathway: under acute hypoxia, FXR signaling promoted lipogenesis and suppressed fatty-acid β-oxidation; in contrast, during chronic hypoxia (long-duration exposure), reduced FXR activity was associated with decreased lipogenesis and enhanced fatty-acid oxidation (Qi et al., 2025). Across both acute and chronic exposure paradigms, hypoxia-driven remodeling of the microbiota–bile acid axis appears to support lipoprotein metabolism, thereby contributing to maintenance of lipid metabolic balance under hypoxic conditions (Yang et al., 2025). Collectively, these findings implicate the gut microbiota and its metabolites as important mediators of adaptation to HAH.

At the level of chronic adaptation, high-altitude native populations exhibit distinct metabolic remodeling strategies. For example, in Tibetan populations, certain variants in EPAS1 (encoding HIF-2α) and PPARα have been reported to associate with reduced fatty-acid oxidation capacity, potentially lowering oxygen demand by restraining lipid oxidation and thereby enhancing hypoxia tolerance (Murray et al., 2018). Epidemiological evidence further indicates that the prevalence of obesity, type 2 diabetes, and coronary heart disease is lower in high-altitude residents than in lowland populations (Hurtado et al., 2012). This phenomenon may reflect, on one hand, long-term hypoxia-associated reprogramming of energy metabolism and changes in body composition (e.g., reduced fat mass), and on the other hand, population-specific genetic adaptation. Notably, a large multi-omics study reported that, among lowlanders who migrated to high altitude, the gut microbiota composition gradually shifted toward that of high-altitude natives, accompanied by corresponding changes in plasma metabolomic profiles; metabolites such as lactate, sphingosine-1-phosphate, taurine, and inositol were significantly increased in the high-altitude environment (Qi et al., 2025). These findings further support the concept that high-altitude hypoxia adaptation involves coordinated, multisystem changes, including remodeling of the microbiota–host co-metabolic network.

However, the metabolic consequences of high-altitude hypoxia are not uniform across populations or exposure contexts. In lowlanders who lack prior acclimatization, prolonged exposure to altitude may induce unfavorable metabolic alterations, including dyslipidemia, inflammatory activation, and other features consistent with increased cardiometabolic stress. By contrast, high-altitude native populations may exhibit population-specific metabolic phenotypes shaped by long-term residence, developmental influences, and evolutionary adaptation. Importantly, these responses should not be interpreted through a single regional lens. Tibetan, Andean, and other high-altitude populations differ in physiological traits such as hemoglobin regulation, ventilatory responses, and likely metabolic strategies, which may in turn influence lipid handling and cardiometabolic risk. In addition, the available literature remains highly heterogeneous with respect to altitude level, exposure duration, nutritional status, physical activity, and baseline metabolic health, all of which complicate direct comparison across studies.

**Figure 1:**
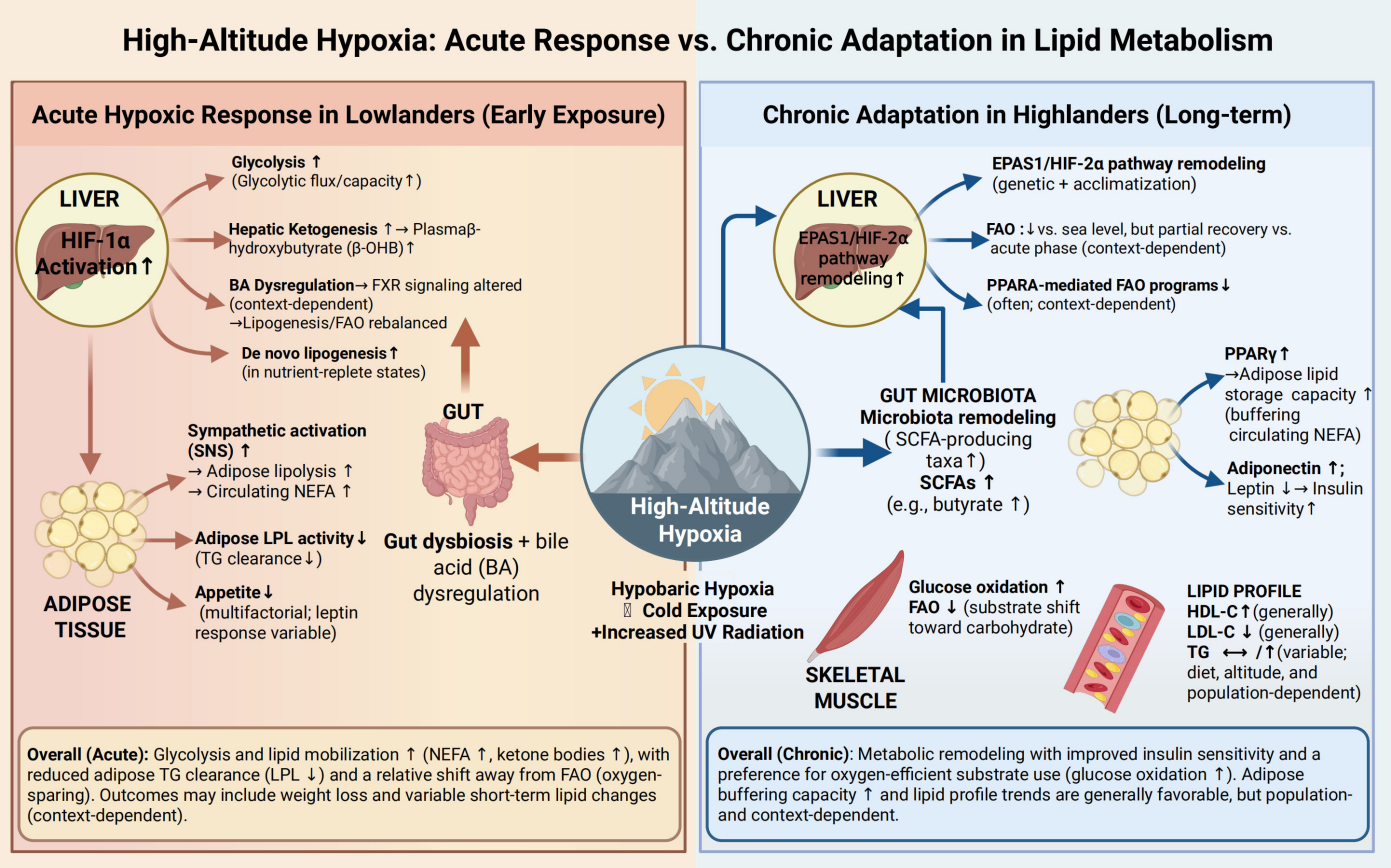
Divergent effects of acute hypoxia and chronic high-altitude adaptation on systemic lipid metabolism. The schematic illustrates the organ-specific metabolic reprogramming triggered by high-altitude environments. (Left) Acute Hypoxic Response: Initial exposure in lowlanders is characterized by sympathetic nervous system (SNS) activation, leading to robust adipose lipolysis and elevated circulating non-esterified fatty acids (NEFAs). HIF-1α activation shifts hepatic and muscular metabolism toward glycolysis, while reduced lipoprotein lipase (LPL) activity impairs triglyceride (TG) clearance. (Right) Chronic Adaptation: Long-term residents or highlanders exhibit metabolic rebalancing. Genetic and epigenetic remodeling of the EPAS1/HIF-2α and PPARα pathways optimizes oxygen-efficient glucose oxidation while modulating fatty-acid oxidation (FAO). This phase is typically associated with improved insulin sensitivity, increased adiponectin, and a more favorable lipid profile, characterized by elevated HDL-C and stabilized LDL-C. The gut microbiota–bile acid axis further contributes to these adaptations through short-chain fatty acid (SCFA) production and farnesoid X receptor (FXR) signaling.

Against this background, a clearer conceptual framework is needed to distinguish acute hypoxic exposure, acclimatization, and long-term adaptation when interpreting lipid metabolic responses to altitude. This narrative review therefore synthesizes current evidence on how high-altitude hypoxia influences lipid metabolism across different exposure stages and population settings, with particular attention to the contrasting patterns observed in high-altitude natives and lowlanders exposed to altitude. We further summarize the principal molecular and systemic mechanisms involved, including HIF-related metabolic reprogramming, neuroendocrine regulation, genetic and epigenetic influences, and the emerging role of the microbiota–bile acid axis. By integrating these perspectives, this review aims to provide a more balanced framework for understanding the heterogeneity of lipid metabolic responses to hypoxia and their implications for future research and clinical translation. A schematic summary of the acute responses and chronic adaptations of lipid metabolism to high-altitude hypoxia is shown in **Figure 1**. These apparently divergent metabolic responses may also be understood within a hormetic framework, whereby appropriately dosed or well-tolerated hypoxic exposure may induce adaptive benefits, whereas more severe, prolonged, or poorly tolerated hypoxic stress may become maladaptive (Burtscher and Samaja, 2024).

## 2 Lipid metabolic traits in high-altitude populations: shared and population-specific features

High-altitude adaptation is not physiologically uniform across human populations. Tibetan and Andean highlanders exhibit distinct patterns of adaptation to hypoxia, suggesting that metabolic traits at altitude should not be generalized from a single regional population (Beall, 2000).

### 2.1 Fatty-acid metabolism and energy substrate utilization in high-altitude adapted populations

High-altitude hypoxia (HAH) imposes distinct constraints on whole-body energy metabolism. Through long-term evolutionary adaptation, high-altitude native populations have developed substrate utilization patterns that differ from those of low-altitude populations. In general, these adapted groups exhibit downregulation of fatty-acid oxidation pathways alongside enhanced glucose oxidation and glycolytic flux. Such a shift can increase ATP production per unit of oxygen consumed, thereby buffering potential energy deficits under hypoxic conditions (Ge et al., 2012; Ren et al., 2023).

In Sherpa populations of the Himalayas, for example, skeletal muscle fatty-acid oxidative capacity is markedly reduced compared with lowland controls; nevertheless, Sherpas demonstrate higher oxygen utilization efficiency, better muscle energetic status, and lower levels of oxidative stress. This phenotype is thought to be partly attributable to selective enrichment of metabolism-related genetic variants. Specifically, Sherpa and Tibetan populations show a higher allele frequency of favorable variants in peroxisome proliferator-activated receptor-α (PPARα; PPARA), which has been proposed to reduce reliance on fatty acids as the primary fuel source in skeletal muscle. In parallel, particular haplotypes of EPAS1 (encoding hypoxia-inducible factor-2α [HIF-2α])—a key locus implicated in high-altitude adaptation—are significantly associated with higher lactate levels, suggesting enhanced glycolytic activity to meet energy demands under hypoxic stress (Ge et al., 2015; Horscroft et al., 2017). Taken together, these genetic and phenotypic data support a coherent model in which adaptation to HAH involves reprogramming of energy metabolic pathways, favoring carbohydrate-based ATP production while relatively suppressing fatty-acid oxidation to satisfy tissue energetic requirements when oxygen availability is limited (Ge et al., 2012; Ren et al., 2023).

### 2.2 Lipid profiles and cholesterol metabolism in high-altitude adapted populations

Circulating lipid levels and cholesterol metabolism in high-altitude adapted populations are shaped by multiple interacting factors, including genetic background, dietary patterns, and environmental exposures. Several cross-sectional studies have reported a non-linear, inverted U-shaped relationship between altitude and lipid indices among Tibetans and other high-altitude residents: total cholesterol (TC) and low-density lipoprotein cholesterol (LDL-C) tend to be higher at moderate altitudes (approximately 2,500–3,500 m) but decline at higher altitudes (>3,500 m). In the Gyarong Tibetan population, for instance, the proportion of dietary energy derived from fat increases with altitude, yet lipid levels still follow an inverted U-shaped trajectory, peaking at moderate altitude and decreasing at higher altitude (Xiaoyue et al., 2023). This pattern may relate to higher energy expenditure under more extreme conditions, reduced appetite, and other altitude-dependent behavioral or physiological changes.

Although traditional high-altitude diets are often rich in yak/mutton and dairy products—implying relatively high saturated fat intake—some high-altitude regions do not exhibit a correspondingly lower cardiovascular disease risk. On the contrary, epidemiological data suggest that dyslipidemia prevalence in certain high-altitude populations may even exceed national averages. Among Tibetan adults in Chamdo, Tibet, the prevalence of dyslipidemia reached 42.7%, substantially higher than the contemporaneous prevalence in the general Chinese adult population (34.0%); the most prominent abnormalities were low high-density lipoprotein cholesterol (HDL-C) and hypertriglyceridemia (Zhang et al., 2012; Nie et al., 2025). Studies from the Andean highlands similarly indicate that children at high altitude are more likely to show dyslipidemia features such as elevated triglycerides and low HDL-C, and their TC levels are significantly higher than those of age-matched low-altitude peers (Hirschler et al., 2012, 2019). Evidence from Andean populations further highlights the heterogeneity of altitude-related lipid phenotypes. In genotype-controlled Indigenous Kiwcha populations from Ecuador, high-altitude residents exhibited distinct hematological adaptation together with higher cholesterol, HDL, and LDL levels than their low-altitude counterparts, although these differences were not accompanied by a clear increase in cardiovascular risk (Ortiz-Prado et al., 2021). These findings suggest that Andean high-altitude residence may be associated with a lipid profile that differs not only from lowland populations but also from patterns described in Tibetan cohorts (Beall, 2000; Ortiz-Prado et al., 2021). Accordingly, the metabolic consequences of long-term hypoxic residence should be interpreted within a population-specific framework rather than assumed to be uniformly protective across all high-altitude groups.

Mechanistically, sustained activation of HIF-1α under chronic hypoxia may promote macrophage uptake of oxidized low-density lipoprotein (ox-LDL), increase synthesis of cholesterol and triglycerides, and inhibit cholesterol efflux, thereby driving intracellular cholesterol accumulation. Accordingly, for individuals without adequate adaptation, prolonged exposure to HAH may increase atherosclerotic risk (Thomas et al., 2022).

Importantly, high-altitude indigenous groups may possess relative metabolic protection. Under comparable high-altitude conditions, Tibetan residents have significantly lower serum triglycerides, TC, and LDL-C than lowland Han migrants living in the same environment (Ge et al., 2015). In school-aged populations in Lhasa, the prevalence of dyslipidemia is also lower among Tibetan adolescents than among Han peers residing at the same altitude (Nie et al., 2025). These observations suggest that population-specific genetic backgrounds and lifestyle factors (e.g., higher habitual physical activity) may partially offset the adverse effects of sustained hypoxia and high-fat dietary patterns on lipid metabolism, conferring a relatively favorable lipid profile in high-altitude natives.

### 2.3 Adipose tissue function and adipokine secretion in high-altitude adapted populations

High-altitude hypoxia not only alters systemic energy metabolism but also reshapes adipose tissue function and its endocrine characteristics, including adipokine secretion profiles. During acute high-altitude exposure, reduced appetite and weight loss are frequently observed; these changes are partly attributed to HIF-1α–mediated upregulation of leptin. Elevated leptin can suppress appetite and increase basal metabolic rate, thereby augmenting energy expenditure (Wang et al., 2007). In contrast, in populations with long-term high-altitude adaptation, leptin secretion appears to shift in the opposite direction. For example, serum leptin levels in native women living at high altitude are significantly lower than those in low-altitude controls, and this difference persists even after adjustment for body mass index (BMI). Moreover, the association between leptin and BMI is attenuated in high-altitude women, suggesting lower leptin secretion at comparable fat mass. This pattern may reflect greater leptin sensitivity and appetite-regulatory mechanisms that differ from those observed in lowland populations (Cheng et al., 2023).

Adiponectin, another key adipokine, enhances insulin sensitivity and promotes fatty-acid oxidation. Evidence indicates that long-term exposure to moderate high altitude may increase plasma adiponectin levels or raise the adiponectin-to-leptin ratio, thereby improving adipose tissue function and systemic insulin sensitivity. High-altitude adapted populations often display a hormonal profile characterized by “relatively higher adiponectin and relatively lower leptin,” which may help preserve insulin sensitivity and reduce susceptibility to metabolic dysregulation.

In addition, low temperature and hypoxia may jointly drive adipose remodeling and activation of brown adipose tissue (BAT). In healthy adults living at high altitude, PET imaging studies have detected metabolically active BAT, with higher activity than that observed in low-altitude populations. One report suggested that hypoxia may suppress the secretion of specific microRNAs from white adipose tissue (e.g., miR-210 and miR-92a), thereby relieving inhibitory constraints on brown fat thermogenic function and enhancing non-shivering thermogenesis (Zhang et al., 2020; Wu et al., 2025). This mechanism has been proposed as an important adaptive strategy under high-altitude hypoxic conditions. BAT activation not only supports thermoregulation in cold environments but can also increase energy expenditure and help counteract excessive lipid accumulation (Li et al., 2018).

## 3 Acute high-altitude exposure and changes in lipid metabolism

### 3.1 Acute shifts in energy substrate utilization

During acute exposure to high-altitude hypoxia (HAH), the profile of energy substrate utilization can change substantially. Experimental data indicate that, under postprandial intermittent hypoxia, the peak rise in plasma glucose is blunted and the increase in insulin is smaller, whereas lactate levels rise markedly. Compared with normoxia, postprandial glycemic responses are attenuated without a clear compensatory increase in insulin secretion, while blood lactate concentrations increase significantly, collectively suggesting enhanced skeletal muscle glucose uptake and increased anaerobic glycolytic activity (Kelly et al., 2010).

Concurrently, postprandial plasma non-esterified fatty acids (NEFAs) are significantly higher under hypoxia than under normoxia, indicating intensified mobilization of lipid stores and greater reliance on lipolysis-derived substrates for energy provision. Mechanistically, acute hypoxia can sharply reduce lipoprotein lipase (LPL) activity in adipose tissue—by up to sixfold in cellular experiments (Mahat et al., 2016). In parallel, sympathetic nervous system activation and increased secretion of stress hormones (e.g., catecholamines) occur, which can further stimulate adipose tissue lipolysis and increase the release of NEFAs into the circulation (Kelly et al., 2010). In addition, acute hypoxia may increase hepatic fatty-acid oxidation rates, leading to higher circulating ketone body levels. Mauger and colleagues reported that, in both fasting and fed states, acute hypoxia tended to increase the production and accumulation of ketone bodies such as β-hydroxybutyrate (β-OHB) (Marcoux et al., 2022). Collectively, in the early phase of high-altitude exposure, the organism appears to enhance glycolysis to secure rapid ATP generation while simultaneously mobilizing lipid oxidation to sustain energy supply in response to an acute oxygen-limited stressor.

From a regulatory perspective, stabilization and activation of hypoxia-inducible factor-1α (HIF-1α) is considered a key driver of these substrate shifts. HIF-1α upregulates glycolysis-related enzymes and suppresses mitochondrial fatty-acid oxidative pathways (e.g., by reducing PPARα-mediated β-oxidation capacity), thereby promoting a shift from lipid-derived fuels toward greater dependence on glucose-based energy production (Horscroft and Murray, 2014). Moreover, because glucose oxidation yields approximately 12% more ATP per mole of oxygen consumed than fat oxidation (i.e., higher ATP production efficiency per unit oxygen), preferential carbohydrate utilization is often viewed as an adaptive strategy to improve energetic efficiency under constrained oxygen supply. These mechanisms provide a plausible explanation for the observed augmentation of carbohydrate metabolism during acute hypoxia.

Notably, a systematic review suggested that, at the same relative exercise intensity, short-term hypoxia does not significantly alter the relative contributions of carbohydrate and fat to energy provision (mean difference in respiratory exchange ratio of only 0.01, p = 0.45). In other words, the impact of acute hypoxia on substrate selection is contingent on factors such as nutritional state and exercise intensity, rather than representing a uniform, unidirectional shift in fuel partitioning (Griffiths et al., 2019).

### 3.2 Short-term fluctuations in circulating lipids

Acute high-altitude exposure can induce dynamic changes in circulating lipid levels. In a controlled trial, healthy men were studied at 490 m and after a gradual ascent to 2,590 m; after two days at 2,590 m, high-density lipoprotein cholesterol (HDL-C) increased significantly, triglycerides (TG) decreased significantly, and the total cholesterol/HDL-C ratio declined markedly. In addition, low-density lipoprotein (LDL) particle diameter increased and LDL subfraction distribution was favorably remodeled (Griffiths et al., 2019). These findings suggest that, during short-term exposure to moderate altitude—particularly in the absence of excess fat intake and in the context of increased physical activity—circulating lipids may transiently improve.

Under different conditions, however, acute hypoxia may increase circulating lipids. In a sustained, fully fed state, hypoxia can impair triglyceride clearance, promoting accumulation of triglycerides carried in very-low-density lipoprotein (VLDL). In a study by Mauger and colleagues, 6 hours of continuous hypoxia significantly prevented the postprandial decline in plasma VLDL-TG and resulted in persistently higher concentrations relative to normoxia (Mauger et al., 2019). This observation is consistent with animal data showing reduced triglyceride clearance under hypoxic conditions, which can precipitate hypertriglyceridemia. Overall, the effects of acute high-altitude exposure on circulating lipids may follow a “biphasic” pattern: with moderate energy intake and some degree of physical activity, lipid levels may decrease modestly in the short term; conversely, under conditions of overfeeding (especially high-fat diets) or sustained energy surplus, hypoxia-associated impairment of lipid clearance may elevate circulating lipid levels.

### 3.3 Effects of acute hypoxia on adipose tissue and endocrine function

Acute hypoxia can also exert pronounced effects on adipose tissue enzymatic activity and endocrine secretion. First, lipid-handling enzymes show rapid alterations: *in vitro* evidence indicates that LPL activity is significantly reduced in human preadipocytes exposed to 3% O_2_. Human studies likewise suggest that brief hypoxic exposure suppresses adipose tissue lipid uptake while enhancing lipid mobilization, thereby increasing plasma NEFA levels. In addition, hypoxia rapidly increases adipocyte secretion of angiopoietin-like protein 4 (ANGPTL4), an endogenous inhibitor of LPL; elevated ANGPTL4 further reduces the capacity of adipose tissue to take up and esterify circulating lipids (Mahat et al., 2016). Acute high-altitude hypoxia also activates the sympathetic nervous system and increases catecholamines (e.g., epinephrine), which likely contributes to the augmented lipolysis and increased NEFA release described above.

At the endocrine level, acute high-altitude exposure can rapidly modify the secretion of adipose-derived hormones. In an acute exposure experiment at approximately 4,300 m, Kelly and colleagues observed a significant reduction in postprandial plasma leptin levels in healthy participants. This change may reflect an acute adipose stress response to hypoxia or suppression of leptin secretion mediated by sympathetic activation. A reduction in leptin could imply a further decrease in lipid oxidation rates, aligning with the broader tendency toward carbohydrate-predominant energy provision during acute hypoxia (Kelly et al., 2010). It should be noted, however, that other studies have reported a transient increase in leptin during the initial phase of high-altitude exposure, potentially contributing to appetite suppression (Pramsohler et al., 2022). This suggests that adipokine responses to acute hypoxia are complex and may vary across individuals, potentially depending on exposure duration, nutritional status, and other contextual factors.

In the study by Kelly and colleagues, the immediate effects of acute hypoxia on other metabolic hormones, including insulin, appeared relatively modest, with no significant changes in insulin levels. Consistently, another study reported no significant effect of short-term exposure to moderate altitude on insulin sensitivity as assessed by the homeostasis model assessment of insulin resistance (HOMA-IR) (Stöwhas et al., 2013). Taken together, acute hypoxia may rapidly elevate stress hormones and downregulate selected adipose-derived factors, promoting a short-term shift away from lipid-based energy provision toward greater reliance on carbohydrate metabolism to accommodate an oxygen-limited environment. A summary of representative studies on acute high-altitude/hypoxic exposure and major lipid-metabolic responses is provided in **Table 1**.

**Table 1:** Representative studies on acute high-altitude/hypoxic exposure and major lipid-metabolic responses.

Study	Population/model	Exposure condition	Feeding/activity context	Main lipid-metabolic findings
Kelly et al., 2010	Healthy humans	Acute altitude exposure (~4,300 m)	Postprandial	Blunted postprandial glucose and insulin responses, increased lactate, reduced postprandial leptin, suggesting enhanced glycolytic flux and altered adipokine responses
Mahat et al., 2016	Human adipose tissue/preadipocytes	Acute hypoxia, including 3% O_2_ in cellular experiments	Not specified	Marked suppression of adipose LPL activity and increased ANGPTL4 response, consistent with reduced lipid uptake and enhanced lipid mobilization
Marcoux et al., 2022	Healthy humans	Acute intermittent and continuous hypoxia	Fasting and fed states	Increased circulating β-hydroxybutyrate tendency under hypoxia, supporting altered fatty-acid oxidation and ketone body production
Mauger et al., 2019	Healthy men	6 h continuous hypoxia	Continuously fed/postprandial	Prevention of the normal postprandial decline in plasma VLDL-TG, indicating impaired triglyceride clearance under acute hypoxia
Griffiths et al., 2019	Meta-analysis/exercise studies	Environmental hypoxia during exercise	Influenced by postprandial state and exercise intensity	No uniform change in substrate partitioning overall; responses vary according to nutritional state and exercise intensity
Stöwhas et al., 2013	Healthy humans	Short-term moderate altitude exposure	Resting/non-training context	No significant short-term effect on HOMA-IR, suggesting limited immediate impact on insulin sensitivity in some settings

Collectively, acute lipid-metabolic responses to hypoxia are highly context-dependent and are influenced by exposure modality, duration, nutritional state, and exercise intensity.

## 4 Long-term high-altitude exposure (non-native individuals): lipid metabolic adaptation

### 4.1 Trajectories of basal metabolic rate and circulating lipid indices

Long-term exposure to high altitude can induce complex metabolic remodeling, with dynamic adjustments in whole-body substrate utilization. Upon initial ascent, tissue hypoxia and heightened sympathetic activity are accompanied by a marked increase in basal metabolic rate (BMR). One study reported that, at an altitude of 4,300 m, BMR increased by approximately 27% relative to sea level in the short term and remained 17% higher even after 10 days (Butterfield et al., 1992). This acute metabolic perturbation increases energy expenditure and promotes the mobilization of lipid stores. With prolonged exposure, individuals gradually acclimatize to hypoxia and the overall metabolic state approaches a new steady state. Metabolomic evidence further supports the view that chronic hypoxic residence in non-native individuals induces progressive systemic metabolic remodeling. In healthy subjects exposed to chronic mild hypoxia for 10 months at the Concordia Station in Antarctica, prolonged hypoxic residence was associated with marked alterations in circulating polar and non-polar metabolomic profiles, including lipid-related pathways. These findings suggest that long-duration hypoxic exposure can reshape systemic metabolism well beyond the acute acclimatization phase and further underscore the metabolic plasticity of lowlanders under sustained hypoxic stress (Dei Cas et al., 2022).

Longitudinal evidence indicates that high-altitude exposure can significantly reshape the lipid profile, although individual lipid parameters do not necessarily change in parallel. In a study by Siqués et al., sea-level residents who lived continuously at 3,550 m for 8 months exhibited a significant increase in triglycerides (TG), a decrease in low-density lipoprotein cholesterol (LDL-C), and relatively stable total cholesterol (TC) (Siqués et al., 2007; Ortiz-Prado et al., 2021). More recently, Wu et al. (2025) reported that, following long-term acclimatization to 2,900 m, high-density lipoprotein cholesterol (HDL-C) increased significantly (Wu et al., 2025). In addition, cross-sectional data in high-altitude native populations suggest that women living at higher altitudes tend to have lower LDL-C but higher HDL-C and TG compared with low-altitude controls.

Overall, long-term high-altitude exposure is frequently accompanied by reduced fat mass and improvements in general metabolic status, alongside adaptive shifts in circulating lipids: TG often increases or fluctuates, LDL-C tends to decrease, HDL-C tends to increase, and TC is comparatively stable. Population-level data in high-altitude residents similarly suggest a relatively favorable lipid profile at higher altitudes (e.g., higher HDL-C and lower LDL-C) (Cheng et al., 2023).

### 4.2 Changes in insulin sensitivity and metabolic health

Multiple studies support a beneficial association between long-term high-altitude exposure and insulin sensitivity. Wu et al. (2025) reported that, after lowland adults relocated to approximately 2,900 m for 12 months, the homeostasis model assessment of insulin resistance (HOMA-IR) decreased significantly, accompanied by reductions in both fasting and postprandial glucose levels (Wu et al., 2025). Consistently, a field intervention study found that 25 days of high-altitude hiking (2,200–3,800 m) significantly improved insulin resistance in individuals undergoing drug rehabilitation, with glycemic regulation returning to a normal range (Lee et al., 2004). Similarly, during a 25-day high-mountain expedition training program, the areas under the curve for both glucose and insulin during an oral glucose tolerance test (OGTT) declined markedly, suggesting enhanced insulin action (Chen et al., 2010).

These metabolic improvements were accompanied by significant reductions in indices of central adiposity (e.g., waist-to-hip ratio), implying that acclimatization may reduce visceral fat accumulation and improve body composition. Epidemiological evidence further links high-altitude residence to better metabolic health: fasting glucose and glycated hemoglobin (HbA1c) levels are significantly lower in high-altitude natives than in low-altitude populations (Cheng et al., 2023), and diabetes and metabolic syndrome prevalence is also lower at higher altitudes. Collectively, in non-native individuals, long-term high-altitude exposure is commonly associated with improved insulin sensitivity—potentially mediated by fat loss and enhanced adipose tissue function—together with broader improvements in metabolic indices such as blood glucose, circulating lipids, and related cardiometabolic parameters (including lower blood pressure, glucose, and LDL-C) (Cheng et al., 2023; Zhou et al., 2023; Wu et al., 2025).

### 4.3 Altered adipose tissue function and regulation of adipokines (leptin, adiponectin)

A substantial body of evidence indicates that non-native individuals often experience significant reductions in body weight and fat mass during prolonged stays at high altitude. A systematic review reported weight loss across most studies, whether exposure was active (e.g., hiking or mountaineering) or passive (resting residence), with greater weight reduction observed when high-altitude exposure was combined with physical activity (Dünnwald et al., 2019). In addition, the hypoxic and cold conditions at high altitude have been proposed to facilitate “browning” of white adipose tissue and thermogenic responses, thereby increasing BMR and enhancing lipid oxidation (Pramsohler et al., 2022).

Recent work further suggests that long-term high-altitude exposure can improve the endocrine function of adipose tissue. Wu et al. reported that, after 12 months of acclimatization at a moderate altitude, serum adiponectin increased significantly while leptin decreased significantly, consistent with improved adipokine regulation and enhanced adipocyte function (Wu et al., 2025). A cross-sectional study in high-altitude residents likewise showed an independent association between higher residential altitude and lower serum leptin concentrations (Cheng et al., 2023). Together, these findings support an adaptive adipokine pattern at high altitude that favors reductions in fat storage: leptin may rise during the early phase of exposure and then decline with acclimatization, whereas adiponectin shows a sustained increase. In combination, these changes may promote energy expenditure and improve insulin sensitivity.

### 4.4 Potential metabolic implications and caveats of long-term high-altitude exposure

Synthesizing available evidence, long-term high-altitude exposure may influence lipid metabolism and overall metabolic health in a context-dependent manner. Although some studies suggest potentially favorable effects on body weight, insulin sensitivity, and selected lipid parameters, these benefits are not uniform across populations or study settings and should be interpreted cautiously in light of major confounding factors such as diet, physical activity, body composition, migration history, and concurrent cold exposure (Dünnwald et al., 2019). Some epidemiological studies have also reported that high-altitude residence is associated with lower obesity risk, lower diabetes prevalence, and improved cardiovascular outcomes. For example, one study reported an inverse association between altitude and obesity risk, with residents at <500 m exhibiting an obesity risk several-fold higher than those living at >3,000 m (Cheng et al., 2023). A large survey in China similarly found that the risk of metabolic syndrome was significantly lower among residents of mid- to high-altitude regions than among those living at low altitude (Zhou et al., 2023).

These putative benefits may reflect integrated regulatory effects of the high-altitude environment on energy balance and lipid metabolism: on the one hand, hypoxia may promote lipid mobilization and oxidation, reducing fat storage; on the other hand, improved insulin sensitivity may further optimize metabolic function. Accordingly, sustained acclimatization to mid-to-high altitude could represent a potentially useful adjunctive strategy for improving chronic metabolic disorders such as metabolic syndrome (Dünnwald et al., 2019; Zhou et al., 2023).

## 5 Exercise and lipid metabolism in high-altitude environments

### 5.1 Changes in substrate utilization during exercise at high altitude

Under high-altitude hypoxia (HAH), the pattern of energy substrate utilization during exercise can shift substantially. It is widely suggested that hypoxia increases the relative contribution of carbohydrate oxidation while constraining fatty-acid oxidation. Indeed, studies of exercise training performed at high altitude or moderate altitude indicate a clear redistribution of energy metabolism. For example, one moderate-altitude exercise study reported that, during both exercise and recovery, the body relied more heavily on carbohydrates than on lipids (Katayama et al., 2010). This shift in substrate preference is closely linked to hypoxia-induced modulation of mitochondrial function and limitations in lipid oxidation capacity (Murray, 2009).

A meta-analysis further noted that the effects of hypoxic exposure on carbohydrate and lipid oxidation during exercise are heterogeneous overall. Importantly, nutritional state and exercise intensity can amplify hypoxia-related changes in substrate selection: under postprandial conditions and at higher exercise intensities, hypoxia is more likely to increase the proportion of carbohydrate oxidation (Griffiths et al., 2019). Mechanistically, hypoxia-inducible factor-1α (HIF-1α) upregulates multiple glycolytic enzymes and pyruvate dehydrogenase kinases (PDK1/4), thereby inhibiting pyruvate dehydrogenase activity and diverting pyruvate toward lactate production (Murray, 2009). In addition, sympathetic activation and enhanced insulin signaling under hypoxia may further facilitate glycolysis. Conversely, hypoxic training can activate skeletal muscle AMPK/GLUT4 signaling, increasing glucose uptake and oxidative capacity (Li et al., 2015). Accordingly, the respiratory quotient (RQ) typically rises during exercise at altitude, consistent with increased carbohydrate utilization and a tendency toward reduced fat oxidation (Murray, 2016).

### 5.2 Effects of altitude training on lipid profiles and body fat

The effects of altitude training on circulating lipids are complex, and results across studies are not fully consistent. Some investigations suggest that altitude training can improve the lipid profile. For example, certain studies observed an upward trend in high-density lipoprotein (HDL) following prolonged altitude training combined with caloric restriction, whereas reductions in triglycerides (TG) and low-density lipoprotein (LDL) appear more dependent on the training protocol and dietary control.

In a 4-week “live high–train low”–type design described as “altitude training–normoxic sleep” conducted under caloric restriction, both the altitude group and the normoxic group showed significant reductions in body weight and fat mass. However, the altitude group did not demonstrate an additional advantage in fat loss, showing only a modestly more favorable trend toward increased HDL compared with the normoxic group (Gao et al., 2020). In another hiking study conducted at moderate altitude, participants exhibited significant reductions in total cholesterol (TC) and LDL-C after 10 days, whereas HDL-C and TG showed no clear changes (Greie et al., 2006). A controlled study comparing 2 weeks of moderate-altitude hiking with low-altitude hiking found that plasma TG and leptin decreased significantly in the moderate-altitude group, while no significant changes were observed in the normoxic group (Gutwenger et al., 2015). Animal data are consistent with a hypoxia-related influence on lipid metabolism: in mice with high-fat diet–induced obesity, 4 weeks of hypoxic endurance training significantly reduced body weight and fat stores (Wang et al., 2019). Moreover, a randomized double-blind controlled trial reported that, among overweight/obese women performing 12 weeks of high-intensity interval training under normobaric hypoxia (FiO_2_ = 17.2%), the hypoxia group achieved greater reductions in body fat percentage and greater increases in lean mass than the normoxia training group, together with improved resting fat oxidation capacity (Camacho-Cardenosa et al., 2018).

Notably, the broader evidence base remains mixed. Recent systematic reviews and meta-analyses have suggested that, relative to intensity-matched normoxic training, hypoxic training can lead to greater weight loss and fat reduction and may provide advantages in improving metabolic indices such as lowering plasma TG and LDL-C (Wang et al., 2024b). In contrast, other studies did not identify clear additive benefits of hypoxic training over normoxic training for lipid profile improvement (Luo et al., 2022). Such discrepancies likely reflect differences in training modality, duration, participant characteristics, and the degree of dietary control across included studies.

Overall, exercise performed under high-altitude (hypoxic) conditions is often accompanied by reductions in body weight and fat mass, but these outcomes are frequently driven by a combination of increased training load and dietary restriction (Dünnwald et al., 2019). Importantly, many altitude-training studies simultaneously implement caloric restriction or high-intensity protocols, making it difficult to disentangle hypoxia-specific effects from the effects of negative energy balance. Well-controlled clinical studies are therefore needed to determine the independent impact of high-altitude hypoxia on lipid indices (e.g., TG, HDL, and LDL) and body fat mass. From a practical perspective, altitude training should not be regarded as a stand-alone strategy for lipid improvement or fat loss. For athletes, hypoxic training may be useful primarily as a performance-oriented training modality, while any metabolic benefits are likely to depend on training intensity, altitude dose, dietary control, and baseline metabolic status. For overweight, obese, or metabolically at-risk individuals, hypoxic exercise may offer potential adjunctive benefits, but current evidence remains insufficient to recommend altitude exposure as an independent therapeutic intervention for dyslipidemia or obesity. Therefore, clinical and training applications should be individualized, and the metabolic effects of hypoxia should be interpreted cautiously in the context of overall energy balance and exercise prescription.

### 5.3 Molecular mechanisms of skeletal muscle metabolic adaptation

Exercise training in a hypoxic high-altitude environment induces a spectrum of adaptive remodeling responses in skeletal muscle. To accommodate sustained oxygen limitation, skeletal muscle engages coordinated regulation of lipid oxidative capacity as well as aerobic and anaerobic metabolic pathways, while integrating broader control of cellular antioxidant defenses and nitrogen metabolism networks to achieve systemic metabolic adaptation (Chicco et al., 2018). In general, environmental hypoxia reduces skeletal muscle dependence on fatty acids and increases reliance on carbohydrates. Clinical and experimental studies indicate that, after high-altitude exposure, skeletal muscle mitochondrial density decreases and fatty-acid β-oxidation is constrained, whereas glucose uptake and anaerobic metabolic capacity are relatively preserved or enhanced (Horscroft and Murray, 2014; Favier et al., 2015).

Interestingly, some evidence suggests that, following acclimatization, the efficiency of long-chain fatty-acid oxidation in human skeletal muscle may improve, potentially via modulation of respiratory chain complex function to sustain lipid oxidation under hypoxic conditions (Chicco et al., 2018). In animal studies, chronic high-altitude hypoxia has been reported to upregulate enzymes involved in skeletal muscle fatty-acid uptake and oxidation (Song et al., 2020). Meanwhile, exercise training itself robustly activates oxidative metabolic programs in skeletal muscle: high-intensity training can markedly increase expression of genes related to glucose transport, lactate transport, and fatty-acid oxidation. However, when the same training is performed under hypoxia, the magnitude of these gene-expression responses appears comparatively attenuated (Drozdovska et al., 2023).

Taken together, the combined influences of high-altitude hypoxia and exercise shift skeletal muscle metabolism toward greater reliance on glycolytic energy provision, while mitochondrial function and fatty-acid oxidation are finely tuned by multiple molecular mechanisms to enable adaptation and compensation under oxygen-limited conditions.

## 6 Mechanisms by which high-altitude hypoxia modulates lipid metabolism

### 6.1 Molecular regulatory mechanisms

Hypoxia can trigger a broad program of adaptive transcriptional reprogramming, shifting cellular metabolism toward lipid accumulation while reducing the flux of fatty-acid β-oxidation. Hypoxia-inducible factors (HIFs) and related metabolic sensors constitute a central regulatory axis in this transition. Sustained hypoxia stabilizes HIF-1α and HIF-2α and drives reprogramming of lipid metabolism; among these, HIF-2α is often considered the dominant mediator of lipid metabolic responses, suppressing fatty-acid oxidation while inducing lipid synthesis and storage. For example, in hepatocytes, HIF-2α downregulates peroxisome proliferator-activated receptor gamma coactivator-1α (PGC-1α) and key β-oxidation enzymes such as carnitine palmitoyltransferase 1A (CPT1A), while upregulating genes involved in lipogenesis, thereby promoting steatosis-like lipid accumulation (Mylonis et al., 2019; Luo et al., 2022).

HIF-1α appears to act synergistically by activating lipogenic pathways. Under hypoxia, HIF-1α (often via PI3K/Akt signaling) induces sterol regulatory element-binding protein 1c (SREBP-1c) and its downstream target fatty acid synthase (FASN), thereby promoting triglyceride (TG) synthesis (Furuta et al., 2008). In addition, HIF-1α can enhance cellular lipid uptake by directly activating transcription of peroxisome proliferator-activated receptor-γ (PPARγ) and upregulating fatty-acid binding proteins (FABP3, FABP4) and lipoprotein receptors (LRP1, VLDLR), increasing the influx of non-esterified fatty acids and lipoproteins (Mylonis et al., 2019). Collectively, HIF-driven signaling promotes preferential sequestration of lipids into lipid droplets and favors glycolytic energy production over fatty-acid oxidation.

The peroxisome proliferator-activated receptor (PPAR) family integrates multiple lipid metabolic signals, and its activity is modulated by hypoxic states. A representative example is HIF-2α–mediated suppression of hepatic PPARα, a key transcription factor governing mitochondrial β-oxidation. In models of hepatocellular steatosis and non-alcoholic fatty liver disease (NAFLD), hypoxia-induced HIF-2α suppresses PPARα, leading to downregulation of fatty-acid oxidation genes such as CPT1A and ACOX1/2 while upregulating lipogenic genes, thereby aggravating intracellular lipid accumulation (Chen et al., 2019; Wu et al., 2022). Consistent with this concept, PPARA variants carried by Tibetan high-altitude populations have been associated with altered plasma non-esterified fatty acid levels (Ge et al., 2012), suggesting evolutionary pressures that favor reduced PPARα-mediated fatty-acid oxidation at high altitude. In contrast, in adipose tissue, HIF-1α can activate PPARγ and promote TG synthesis and storage, underscoring tissue-specific differences in PPAR-mediated regulation (Mylonis et al., 2019).

SREBP-1c and downstream lipogenic enzymes are robustly upregulated under hypoxia. Hypoxia can activate SREBP-1c (partly via Akt- and HIF-1α–dependent mechanisms), enabling it to bind promoters of lipogenic genes such as FASN (Paschetta et al., 2015) and directly enhance *de novo* fatty-acid and TG synthesis. In fact, tumor cells and hepatocytes in hypoxic environments often exhibit strong HIF-dependent induction of SREBP-1c and FASN, and this lipogenic amplification is considered important for cellular survival under oxygen limitation (Mylonis et al., 2019). Accordingly, hypoxia shifts hepatocyte and adipocyte metabolism toward lipogenic programs.

With respect to fatty-acid oxidation, integrated HIF signaling broadly suppresses β-oxidation (Du et al., 2017; Mylonis et al., 2019). HIF-1α and HIF-2α jointly inhibit expression of key β-oxidation machinery—including CPT1A and multiple acyl-CoA dehydrogenases—through downregulation of PGC-1α/β, resulting in significant decreases in enzymes such as CPT1A, medium-chain acyl-CoA dehydrogenase (MCAD), and long-chain acyl-CoA dehydrogenase (LCAD) under hypoxia. This regulatory pattern forces cells to rely more heavily on anaerobic glycolysis for ATP production. Animal studies similarly show that chronic hypoxia (e.g., intermittent hypoxia exposure in mice) reduces expression of mitochondrial β-oxidation–related enzymes (Mylonis et al., 2019; Reddan and Cummins, 2025).

In parallel with suppression of oxidation, hypoxia activates pathways for lipid uptake and storage. Hypoxia upregulates fatty-acid binding proteins (FABP3, FABP5, FABP7) and the lipid transporter/receptor CD36, promoting uptake and utilization of exogenous fatty acids (Bensaad et al., 2014; Mylonis et al., 2019; DeBerge et al., 2024). HIF-1α can also directly regulate rate-limiting enzymes in TG synthesis, including AGPAT2 and Lipin-1 (Triantafyllou et al., 2018). Moreover, HIF signaling induces lipid droplet–associated proteins such as PLIN2 (perilipin-2) and HILPDA (also known as HIG2), stabilizing neutral lipid droplets and suppressing lipolysis (Xiao et al., 2024; Huang et al., 2025). The net effect is the formation and accumulation of abundant cytosolic neutral lipid droplets under hypoxia, which may both mitigate fatty acid–induced lipotoxicity and provide a readily mobilizable energy reserve during re-oxygenation. Nitric oxide-related pathways also merit consideration in the metabolic response to high-altitude hypoxia. Nitric oxide (NO) is a key component of altitude acclimatization and long-term adaptation, with population studies showing that Tibetan highlanders differ from Andean highlanders in NO-related physiology. Beyond its vascular effects, NO can modulate mitochondrial respiration and cellular substrate utilization, thereby influencing how oxygen availability is matched to metabolic demand. Although direct evidence linking NO signaling to altitude-related lipid remodeling remains limited, differences in NO handling may contribute to population-specific variation in lipid metabolic phenotypes and cardiometabolic risk under hypoxic conditions (Beall et al., 2012).

### 6.2 Neuroendocrine regulation

Acute high-altitude hypoxia rapidly activates the sympathetic nervous system, increasing circulating norepinephrine and elevating blood pressure and heart rate (Hainsworth et al., 2007). Sympathetic activation enhances adipose TG breakdown via β-adrenergic receptor signaling, releasing large amounts of non-esterified fatty acids (NEFAs) into the circulation. These fatty acids are delivered to the liver as substrates that can support very-low-density lipoprotein (VLDL) synthesis. Concurrently, sympathetic activation suppresses insulin secretion and weakens insulin-mediated restraint on adipose tissue, further amplifying lipolysis (Morin et al., 2021). High-altitude hypoxia can also increase cortisol levels (Tsunekawa et al., 2022); cortisol promotes hepatic gluconeogenesis and may indirectly support lipid mobilization while helping maintain glycemic stability.

During the acute hypoxic phase, some studies have reported a significant rise in serum leptin levels (Rausch et al., 2018), associated with appetite suppression and accompanied by enhanced fatty-acid oxidation (Pramsohler et al., 2022; Wang et al., 2024a). Reduced food intake, together with increased lipid mobilization, contributes to early weight loss during initial exposure. As exposure progresses into longer-term acclimatization, these neurohormonal responses tend to rebalance, with compensatory endocrine adjustments emerging.

Evidence indicates that insulin sensitivity can improve during mid- to long-term high-altitude exposure (Koufakis et al., 2019). For example, in a 12-month exposure study at moderate altitude (approximately 2,500–3,000 m), fasting glucose and HOMA-IR decreased, serum adiponectin increased, and leptin decreased. These findings suggest that chronic hypoxic exposure may improve adipose tissue function and insulin signaling, supporting metabolic homeostasis. In addition, high-altitude adapted populations often display lipid profile features such as higher HDL and lower TG, which may relate to improved insulin sensitivity and increased adiponectin (Yanai and Yoshida, 2019; Wu et al., 2025). Overall, the sympathetic–endocrine axis appears to respond in the acute phase primarily by promoting lipid mobilization and thermogenesis to meet energetic needs, whereas during longer-term acclimatization it contributes to dynamic metabolic balance through adjustments in insulin, leptin, and related hormonal signals.

### 6.3 Genetic and epigenetic regulation

High-altitude populations exhibit clear genetic signatures of adaptation relevant to lipid metabolism. Genomic studies in Tibetan and other eastern Himalayan high-altitude populations have identified loci such as EPAS1 (encoding HIF-2α) and PPARA (encoding PPARα) as closely associated with lipid metabolic phenotypes. Individuals carrying adaptive EPAS1 alleles show significantly higher blood lactate levels, consistent with greater glycolytic activity; in parallel, an increased number of specific PPARA haplotypes is associated with higher serum NEFA concentrations, implying reduced fatty-acid oxidation capacity (Ge et al., 2012; Murray et al., 2018). These data support the hypothesis that, under high-altitude hypoxia, metabolic fuel preference shifts from fatty-acid oxidation toward glucose metabolism to reduce oxygen demand. As a key transcriptional regulator of fatty-acid β-oxidation, PPARα variants may alter the functional efficiency of mitochondrial β-oxidation pathways, thereby affecting lipid handling and utilization. In addition, high-altitude–specific EGLN1 variants (encoding prolyl hydroxylase domain protein 2) can enhance prolyl hydroxylase activity and reduce sensitivity of the HIF pathway to hypoxia, influencing cellular responses to hypoxic signaling; however, the specific implications for lipid metabolism remain to be clarified.

Andean high-altitude populations also show adaptive variation related to EPAS1 (HIF2A) (e.g., variants associated with high-altitude erythrocytosis), and it has been proposed that such variants may facilitate metabolic acclimatization by altering the balance of substrate utilization (Lawrence et al., 2024).

At the epigenetic level, hypoxia can exert profound effects on lipid metabolism via mechanisms including DNA methylation and microRNAs (miRNAs). High-altitude mountaineering experiments have shown that short-term hypoxic exposure increases DNA methylation within the PPARα promoter region. Given that DNA methylation generally suppresses transcription, this change is consistent with observed reductions in PPARα expression under hypoxia (Childebayeva et al., 2019), thereby lowering β-oxidation flux and shifting energy metabolism toward glycolysis. Additional evidence suggests that, under high-altitude conditions, expression of the miR-210/92a cluster decreases in exosomes secreted by white adipose tissue, relieving inhibition of fibroblast growth factor receptor 1 (FGFR1) signaling in brown adipose tissue (BAT) during hypoxic stress and promoting BAT activation and thermogenesis (Zhang et al., 2020). Conversely, miR-210—a prototypical “hypoxia miRNA (hypoxamir)”—is upregulated in certain models and can inhibit brown adipocyte differentiation and thermogenic capacity (Caca et al., 2024). Together, these miRNA-mediated mechanisms contribute to hypoxia-driven reprogramming of tissue energy metabolism.

In summary, hypoxia-induced epigenetic changes—such as increased methylation of the PPARα promoter and remodeling of adipose tissue miRNA profiles—can regulate lipid metabolism–related genes and signaling pathways, helping optimize energy utilization and thermogenic function to adapt to the oxygen-limited high-altitude environment.

## 7 Conclusion

High-altitude hypoxia exerts context-dependent effects on lipid metabolism that vary according to exposure duration, acclimatization status, population background, and baseline metabolic health. On the one hand, hypoxia-driven metabolic reprogramming—largely orchestrated by hypoxia-inducible factor (HIF) signaling—shifts energy production away from fatty-acid oxidation toward more oxygen-efficient substrate utilization, thereby supporting adaptation under oxygen-limited conditions (Mylonis et al., 2019). With prolonged exposure, progressive acclimatization may be accompanied by adaptive endocrine and metabolic remodeling, including lower leptin levels, higher adiponectin levels, improved insulin sensitivity, and, in some settings, a relatively favorable lipid profile (Zhou et al., 2023; Wu et al., 2025).

On the other hand, in individuals who are not adequately acclimatized, sustained high-altitude exposure may precipitate lipid metabolic dysregulation and adverse cardiometabolic consequences, including dyslipidemia, inflammatory activation, and features consistent with a pro-atherogenic metabolic phenotype (Pooja et al., 2021). These findings indicate that alterations in lipid metabolism under HAH may represent either an oxygen-conserving adaptive strategy or a source of cardiometabolic vulnerability, depending on individual susceptibility and the specific exposure context.

In addition to HIF-mediated transcriptional reprogramming, emerging evidence highlights important contributions from the gut microbiota–bile acid axis (Yang et al., 2025), neuroendocrine signaling, genetic adaptation, epigenetic regulation, and nitric oxide-related pathways. Together, these mechanisms help explain why lipid metabolic responses to hypoxia are heterogeneous across populations and exposure stages rather than uniformly beneficial or detrimental.

Several limitations of the current evidence base should be acknowledged. First, this article is a narrative review rather than a systematic review, and the available literature remains highly heterogeneous with respect to altitude level, exposure duration, nutritional status, physical activity, study design, and metabolic background. Second, important confounding factors—including diet, body composition, migration history, and environmental co-exposures such as cold—are not consistently controlled across studies. Third, the current literature remains geographically imbalanced, with substantial representation of Tibetan and Asian cohorts but comparatively limited evidence from Andean, African, and other high-altitude populations. Finally, much of the mechanistic evidence derives from animal or cellular models, and direct causal links between specific hypoxia-related pathways and human lipid metabolic phenotypes remain incompletely defined.

From a clinical and translational perspective, these findings suggest that lowlanders newly exposed to altitude, individuals with obesity or dyslipidemia, and other metabolically vulnerable populations may require closer monitoring during prolonged hypoxic exposure. At the same time, the potentially beneficial metabolic effects of acclimatization or altitude-related interventions should not be overstated, particularly in the absence of careful control for diet, exercise, and energy balance. Future studies should prioritize well-controlled longitudinal designs, more balanced inclusion of Andean and other underrepresented high-altitude populations, and integrated multi-omics approaches to clarify how hypoxia modifies lipid metabolism across different human populations and exposure scenarios.
